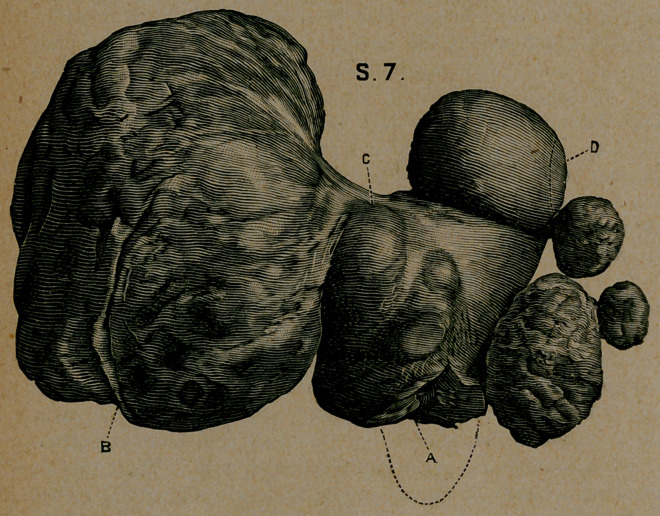# Recent Cases of Cœliotomy

**Published:** 1893-02

**Authors:** A. E. Spohn

**Affiliations:** Corpus Christi


### DANIEL'S TEXAS MEDICAL JOURNAL.

ESTABLISHED JULY, 1885.

PUBLISHED MONTHLY.—SUBSCRIPTION $2.00 A YEAR.

Vol. VIII. AUSTIN, FEBRUARY, 1893. No. 8.

### Original Contributions.

For Daniel's Texas Medical Journal.

### RECENT CASES OF CŒLIOTOMY.

BY A. E. SPOHN, M. D., CORPUS CHRISTI.

[Read at Waco, at joint session Austin District Medical Society and Central Texas Medical Society.]

I HAVE selected this subject for your consideration this evening, being rather a report of my experience in abdominal surgery at the Bay View Sanitarium in Corpus Christi, Texas, during the past few months.

It is impossible to estimate the recent advancement, or possibilities of excellence, which may be attained in this branch of surgery, in the near future. Such has been the success, that diseased conditions heretofore considered incurable, are now being treated rationally and successfully, thereby materially reducing the number of invalid women in our communities. We might naturally ask, why such brilliant results have been reached. Is it because we treat our patients differently, or any change of technique in our methods? I think not; but that we appreciate pathological conditions more fully, and realize the fact that a lesion, or diseased condition, wherever found, should be remedied, the part restored to its normal state, or removed, as a useless and disturbing portion of the economy.

In these days of advancement, the general practitioner can no longer relegate all his unfortunate cases to the care of a specialist; he is compelled to be prepared to meet certain emergencies, be it a case of Cæsarean hysterectomy, acute septic peritonitis or ectopic pregnancy. Rare as they are, they do occasionally occur, and it has been my fortune, and misfortune, to have seen several such cases recently; and if occurring in the practice of one located in a small town surrounded by a thinly populated district, they must certainly be much more frequent in large cities.

There is no case more tests the skill,
The steady nerve, and power of will,
Than where, from causes now unknown,
A living ovum has been thrown
Into the tube, is fixed and grows,
And by increasing slow, so slow,
That tube no longer can contain
Its living contents, bursts, in twain is rent,
The life blood flows from unclosed vent,
The mother sinks, her eyes grow dim,
All hope seems lost. Hold ! See, comes in
A noble mind, the bravest heart,
A steady hand well trained in art;
He ope's the wall, a string is laid
Around the tube, her life is saved.
There is no time so sure we stand,
Holding a fellow life in hand,
Decision, acting well our part,
Steady of nerve and brave at heart,
Doing no more than should be done
To our own selves, were we the one.

Before giving a report of special cases, I will give a resume of my method of making an abdominal section. Not that it differs much from that of other surgeons, still there are certain steps I think peculiar to myself; or rather, I have selected what I have chosen to term a rational method,—no reason without a cause, no cause without a reason,—and if you consider my method or treatment worthy of imitation, I shall consider myself more than paid for the trouble in preparing this report.

Having decided it is necessary to open the abdominal cavity, a certain technical preparation of the patient, instruments, assistants and operator is of the greatest importance.

I will presume the operator has selected a well ventilated room in a healthy locality, with good hygienic surroundings; and I think small, private hospitals, outside of crowded cities, much preferable for this work, and will be attended with much better results, where the surgeons are equally skillful, than in densely populated centres. "The safeguard of every community, no matter how situated, is to see to it that it has, and encourages some man by its support to devote himself sufficiently to such study and investigation as will enable him to rise equal to an emergency of this order, with good hope of success."

In preparing a patient for cœliotomy, the most rigid cleanliness must be observed. The room should be aseptic; all bedding, in fact, clothing coming in contact with the patient, should be previously boiled. It is not sufficient to bathe with an antiseptic solution the day of the operation. A daily bath should be given several days before, using green soap over and around the site of the operation, after which the parts are dusted with boric acid. The vagina should also be irrigated daily, with a five per cent. boric acid, or one to three thousand corrosive sublimate solution. All water used should be sterilized by boiling. If there is much secretion coming from the uterus, its cavity should be wiped out carefully with dry aseptic cotton, then cleansed with peroxide of hydrogen, 15 vol. solution, one part to three of water, the vagina wiped dry and dusted with boric acid. If the parts are to be shaved, it should be done at one of the dressings, ostensibly for the purpose of cleansing, and not when under the influence of an anæsthetic. During this preparation, a light, nutritious diet should be given, bowels regulated, and the morning of the operation a dose of salts given, to insure free action of the bowels, and only a little tea or water allowed. The instruments should be carefully selected, to meet any emergency arising, avoiding a useless display, boiled in water containing a little bicarbonate of soda, and placed in a tray of boiling water. I keep my thread, catgut and horsehair, also silk-worm gut ligatures, in straight glass tubes, about two feet long, which always keeps them straight, avoiding delay by twisting and coiling up. The tubes may be filled with some aseptic solution to suit the surgeon. I prefer horsehair to all other ligatures for outside work, and closing incisions, and prepare it myself, as follows: Select long black hair, wash it thoroughly in sterilized water, using green soap. It is then placed in a ten per cent. solution of carbolic acid, or one to two thousand corrosive sublimate solution, for forty-eight hours. Wash again in sterilized water, and place it in a glass tube containing boro-glyceride, fifty per cent. One end of the tube may be sealed, the other closed with a cork. Prepared in this way, the horsehair is strong, quite elastic, and will keep any length of time. I use a strong, round pointed, double edge knife, with aluminum handle, a needle and holder which carries several sizes of thread, being quite useful for quick work. The knife was made by Gemrig & Son, of Philadelphia; the needle by Geo. Tiemann & Co., of New York, from designs I furnished them. I have long since abandoned the use of sponges, using instead aseptic gauze, and when the gauze is to be inserted into the abdominal cavity, the edges should be folded and stitched, or made into pads. All water should be filtered, boiled, and kept in large jars or pitchers, well covered with aseptic cotton; some hot, others cold. And the gauze can be conveniently kept in one of these jars, a good plan being always to use the same number of pieces of gauze, a record of which, together with all instruments used, should be kept. A large fountain syringe should hang convenient to the operating table, with a large metal tube, for washing the abdominal cavity. The tube I use is my own design, made by Gemrig & Son, consisting of a large tube with round conical end, an opening on either side one inch from the end, a slot passing from the opposite sides entirely around the end, allowing the water to flow, not only from the two openings, but also in a broad stream from the sides and end of the tube. All ligatures should be sterilized. Those left within the cavity may be of specially prepared catgut; still, when I use a ligature I wish one with good staying qualities, consequently I prefer silk, and have never known them to give trouble in any of my operations, having in one case of gunshot wound of the abdomen inserted seventy-two stitches, besides several ligatures, where there were nine openings in the intestines, my patient making a quick and uncomplicated recovery. (*Phil. Med. and Surg. Reporter.*)

A drainage tube should be used when there have been many adhesions; there is hemorrhage, or an escape of foreign matter into the cavity: but drainage tubes should be avoided as much as possible. I always leave a temporary drainage tube, passing into the sac of Douglas, until ready to close the abdominal incision, to be sure there is no hemorrhage. In closing the incision I use silk and horse hair, with cat-gut to approximate the muscles or tendon, as buried sutures; the horse hair and silk alternating, and remove the silk first; the horse hair does not irritate, and may be left until the union is quite firm. The line of incision is wiped very dry, then covered with boric acid, using it freely, about one-fourth of an inch thick, extending one inch on either side of the incision. A piece of borated cotton, about two inches wide, is next laid over the incision, extending one inch above and below. This makes a dry absorbent, aseptic dressing; which need not be disturbed until ready to remove the stitches, on the 7th, 8th or 9th day. The next step is to apply the adhesive strips, which I consider quite important, the object being to fix, as it were, and give support to the line of incision. I use strips of good rubber plaster two inches wide, and long enough to extend about four or six inches on either side of the incision, thus fixing and supporting the central line, allowing motion of the abdominal walls on either side. I again dust the surface of the abdomen freely with boric acid, cover with borated, or recently baked cotton, and apply a well-fitting flannel bandage quite firmly. I have never seen failure of union by primary adhesion, under above method, and have never been compelled to change the dressing until ready to remove the stitches. It is in my opinion an ideal dressing. I have never used iodoform, and consider anything useless and dangerous, that will mask an offensive odor by one still more offensive.

Returning to the operator and his assistants, I have only to state that the utmost cleanliness should be observed; in fact a surgeon should not be present in clothing worn during general practice; and at my infirmary I furnish my assistants aseptic linen aprons. The hands should be carefully cleansed, using a brush and green soap, with sterilized water, then a one to one thousand solution of corrosive sublimate, followed by a saturated solution of oxalic acid, wiped dry and washed in alcohol. The vicinity of the incision may be treated in the same manner. During the operation sterilized water is used only. An exceedingly nice operating apron is made as follows: enough of what is known as butcher's linen to reach from the ankles over the shoulder and fall back behind reaching to the waist; corresponding to the neck cut an opening, also extending down the back. A band or narrow collar is fitted to the neck portion, which with the slit down the back is made to button. Tapes are fastened on either side; also to portion dropping over the shoulder, which are tied. Such an apron costs but about 75 cents.

The after treatment is quite simple. I allow the lips to be moistened with water, and sometimes give a little crushed ice, or an occasional teaspoonful of cool water or tea, during the first day, increasing the quantity a little the second day if the stomach is not irritable. The afternoon of the second day I give 1-10th of a grain of calomel, with bicarbonate of soda, every hour, until one-half to one grain has been taken, followed next morning by teaspoonful doses of salts in a little water, every two hours until the bowels act freely. When there is much trouble with flatus I insert a long glass drainage tube into the rectum, and through this tube wash out the bowel. Opiates should be avoided if possible, but since most cases are old sufferers when operation is attempted, accustomed to the use of morphine, it is advisable in such to continue the opiate for a time.

This report includes nine cases of cœliotomy for various causes; two ovarian abscess; one cæsarean hysterectomy, in a malacosteon; one ovarian tumor; one salpingitis with prolapsed adherent ovaries; one fibroid tumor, multinodular, requiring, cœlo hysterectomy; one fibroid tumor in a girl six years of age; one acute septic-peritonitis; one gunshot wound of the liver. I was assisted in the operations by Drs. Heaney, Westervelt and Hamilton, of Corpus Christi, and my students, T. S. Burke and Jno. Westervelt.

Case 1. Mattie L., age 26, married, no children, no miscarriages, no evidence of specific disease, very weak and emaciated. This young woman had been a constant sufferer for three years, previous to the operation. Severe dysmenorrhœa, with almost constant pain over seat of ovaries. I attended her from time to time, being compelled to give morphia for relief. Her temperature ranged from 101° to 103° F., with fever, night sweats and chilly sensations at irregular intervals. The uterus was fixed, left ovary very much enlarged and painful, right very painful and enlarged. I made a cœliotomy on the 1st of November, 1891, and found the parts as I had anticipated. The adhesions were very extensive, and I removed both ovaries and tubes, with difficulty. She made a quick, uncomplicated recovery. Highest temperature, 99½° F. Specimen No. 1 contains her ovaries and tubes. The left ovary is very much enlarged, covered with a mass of adhesions. There is a cyst in the tube containing pus, and under the ovary is quite a large abscess. The right ovary is also enlarged, and the tube is very large, containing pus. I had to tear these ovaries and tubes from behind the broad ligaments, with the greatest difficulty, as may be readily seen from the extensive adhesions. No drainage.

Case 2. Cæsarean hysterectomy in a malacosteon. This case is of unusual importance, being the "first operation for this condition ever performed in the United States." (Dr. Robert P. Harris, Phila.)

Mrs. G., age 42, very short and stout, the mother of nine children. About four and six years ago I attended this woman in childbirth,—both deliveries difficult, instrumental; the last extremely difficult. I then told her she could never give birth to a child again. She is a malacosteon, and from her peculiar position working, kneeling with the body bent forward (a tortillera), her spinal column had curved forward, in the lower dorsal and lumbar regions, until the apex of the curvature was but two and one-half inches from and a little above the pubic arch. On the 20th of November, 1891, I was again called to see her, and was very much surprised to find her again in labor, at full term. She had been in labor three days. Upon examination, I found it impossible for her to give birth to her child, and decided to open the abdomen, and finish by removing the uterus and appendages. The membranes had ruptured. I irrigated the vagina, and as far into the uterus as possible, with sterilized water; also a bichloride solution, 1 to 4000. The surface of the abdomen was also carefully cleansed. She presented quite a peculiar appearance, the head of the child resting above the brim of the pelvis, with the uterus standing prominently out, like a large conical elongated body. I made an incision through the navel, extending well down to the pubes. Upon entering the abdominal cavity, I enlarged the incision sufficiently to allow the fundus of the uterus to protrude a little, which was caught with strong vulsellum and held while I cut directly into it, having a rubber tube ready to tighten around the organ as I drew it out. I next passed two fingers of each hand into the incision in the uterus, and as I drew it out tore it open, and before the uterus was delivered, or as soon as torn sufficiently open, the child was forced out by contractions. The rubber tube was tightened as the uterus came through the abdominal incision, and a long piece of aseptic gauze wound around the uterus to prevent the escape of any of its contents into the abdominal cavity. I made the incision directly into the center of the placental attachment. There was very little hemorrhage. It is surprising how easily the uterus can be torn, and you will see, in specimen No. 2, how extensively I tore it open. I was but two minutes delivering uterus and contents,—a well-developed, living child.

The next step was to secure the pedicle, which was quite difficult, on account of the great thickness of the abdominal wall. I passed four long, steel knitting needles through the pedicle just above the constricting rubber tube; above these I placed a strong ligature, to diminish the size of the pedicle, and support the needles. The rubber tube, being quite small, was passed twice around the pedicle, and tied. I cut the uterus away near the ligature above the needles, and applied actual cautery to the end of the pedicle, using a small copper soldering iron. The pedicle was composed of the round ligaments, tubes, broad ligaments, and a portion of the neck of the uterus. The appendages were removed with the uterus. I had carefully avoided the bladder,

S. 2.

Uterus removed from a malacosteon. Cæsarean hysterectomy. A, neck of uterus; B, round ligament, tube and ovary; C, opening where torn to deliver child, one-fourth natural size.

by keeping a sound in that viscus while removing the uterus. The abdominal cavity was washed out with sterilized water, no drainage tube was used, and the abdominal incision closed with silk, the stitches near the pedicle passing through it just below the constricting tube of rubber. The incision was dressed with boric acid, and pads of borated cotton placed under the knitting needles. The pedicle came away on the tenth day, leaving a continuous opening between the vagina and abdominal incision, which gradually closed by granulation, and in thirty days she was quite well.

This woman had no pain after the operation, did not know for fifteen days how her child had been delivered, rested well, and had no more trouble than after an ordinary labor. Her child was healthy and strong. I feared, at first, it was injured from pressure against the apex of the curvature, which had made a deep indentation into the child's head, as if pressed in by a hard substance as large as an orange. She did not nurse her child, for some cause, having very little milk, the same condition existing with previous children. It is now nearly a year since the operation. Mother and child are quite well, and she still makes her living grinding tortillas. I met her on the street a few days ago, carrying a sack of corn on her head, when she stated she was well and strong, looking very little like a malacosteon who had undergone an operatisn for cœlo-hysterectomy.

CASE 3. M. C., single, age 18. In September, 1891, her family noticed she was getting large; her menses had ceased for several months. I examined her and found quite an enlargement in the abdominal cavity and had some difficulty in determining the cause of her trouble. I made a careful examination, while under chloroform, and through the rectum could easily outline the uterus. On the 6th of April, 1892, I made a cœliotomy, removing a large multilocular ovarian tumor, right side. The left ovary was enlarged; size of a lemon, with commencing cystic degeneration. I removed it also. She made a good re-

S. 3.

Cystic degeneration of ovary, showing large cyst laid open, with other smaller cyst projecting. One-third natural size, covery. The only interest in this case is the pathological specimen No. 3, commencing cystic degeneration of an ovary, in which may be seen numerous small cysts. [Since reporting this case I have been consulted by this girl for an abscess near line of incision, which I opened, and found a sinus, which, believing it was caused by a ligature, I tried to remove. I passed a probe made of doubled horse hair into the sinus, turning it occasionally; at the end of a few hours the probe was withdrawn with the ligature caught in one of the loops. This little mishap gives me an opportunity of describing a simple yet most effective method for removing ligatures from sinuses. To make this probe I use about 30 aseptic horse hairs, 12 inches in length. They are doubled, and the free ends clamped with a shot; a thread is tied to each end and so fastened as to keep the hair straight; the hair is now wet with aseptic glue, by boiling, twisted a little and dried. When passed into a sinus the glue becomes moist, liberating the hair, thus placing in the vicinity of the ligature 30 loops, which when withdrawn will have the ligature caught in one of them.

CASE 4. Mrs. B., age 31; mother of one child, born August, 1891. This lady has been an invalid for 10 years. Her trouble began after being thrown from a carriage, producing retroversion of the uterus; followed by pain in the left ovarian region, with severe dysmenorrhœa, and great difficulty and pain in having an action from bowels. In 1890, she had influenza, which very much aggravated her ovarian trouble. She first consulted me in March, 1892. I found the ovaries prolapsed and extremely sensitive. She did not complain of pain in adjacent parts, but whenever I touched the region of ovaries, it caused severe paroxysms of pain, so great, that I was compelled to inject morphia. She had gone the usual rounds seeking relief, and I tried, without success, almost everything recommended for such conditions, with rest; and finally considered her case one requiring, at least, an exploratory incision. Upon opening the cavity I found just what I had anticipated, a retroverted uterus; prolapsed and adherent ovaries, both of which I removed. She made a quick recovery, and has been free from pain since, being completely liberated from the distress which followed her as a shadow. Specimen No. 4 contains the ovaries and tubes. In the left is a commencing cyst occupying almost the entire organ, which is covered by adhesions. The right ovary is cirrhotic. In this case, while the right ovary is not much diseased, I feared, if left, her trouble would continue; and believe the relief gained justified the means.

CASE 5. Adela G., age 6 years; well nourished and apparently in good health. This little girl was sent to me by the Laureles Pasture Company. I found quite a large tumor in the abdomen, right side, which I supposed, at first, was a lipoma, probably in the abdominal walls. In attempting to remove the growth, I found it dipped down into the pelvic cavity, and was attached by a pedicle to the right broad ligament, near the uterus, or the uterus itself. The pedicle was ligated and tumor cut away. She made an excellent recovery, and is now apparently quite well. Operation was performed September 10th, 1892. The incision was quite extensive and I was very much troubled by protrusion of the intestines. I wrote to Dr. Robt. P. Harris, of Philadelphia about this case, and he thinks the chances are that the growth is a carcinoma. Specimen No. 5 is the tumor, the nature of which I have not yet determined.

CASE 6. Mrs. D., age 38; one child, 18 years ago, one miscarriage 20 years ago; very weak and anæmic; has been an invalid since birth ot her child; severe pain in lower abdominal region, extending down limbs. Pain was most severe at first in right side and groin, then in left. She has had occasional fevers, with chills; eight weeks ago she had a very severe attack, with pain in lower abdomen, fever and chills, which continue daily. She cannot sit up or walk, and is troubled with night sweats; in fact, a perfect physical wreck. I examined her for the first time on the 15th of September, 1892. Cervix normal, resting against the anterior vaginal wall, high up; uterus fixed; left tube and ovary enlarged, presenting to the touch a nodular condition. In the right side was a hard mass, firmly fixed by adhesions. The uterus was elongated, the fundus carried up behind the pubic arch, and to the right. The anterior wall of the rectum was lifted up, or arched, which led me to believe she had a round growth in the right pelvic region, attached to the rectum and broad ligament, probably an abscess. I was afraid she would die before I could operate, she having been brought three hundred miles by railroad, on a bed. I had to hurry the operation, without preparation, and on the 17th of September made a cœliotomy. Her pulse was now 130, temperature 103° F. Upon opening the abdomen, I found evidences of various attacks of peritonitis, and had much difficulty in reaching the pelvis, being compelled to tear my way through dense adhesions. The right ovary was about the size of a large orange; and the tube thickened to one inch in diameter, firmly bound to surrounding parts by adhesions. In attempting to enucleate the mass, I broke the tube off, it coming away, and looking like a thick piece of wax. I dissected out the ovary by tearing carefully through the adhesions, as it were, enucleating it. The hemorrhage was so severe that I was compelled to ligate the broad ligament on either side of the mass, and after its removal, packed the part firmly with aseptic gauze. I found the same condition on the left side, but succeeded in turning out the mass from behind the broad ligament and ligating the pedicle. A drainage tube was used forty-eight hours. This woman made a good recovery. Speci-

S6

Multinodalar fibroid tumor of uterus. A, neck of uterus; B, omental attachments; C, fundus of uterus; D, line where broad ligament was cut away which included the three small tumors from which peritoneum has been removed. One-sixth natural size.

men No. 6 contains the parts removed, consisting of the two ovaries, about the size of oranges, filled with thick, yellow pus. The tubes are very large, about one inch in diameter, the fimbriated ends pressed against the mass. The tubes contain no pus, simply very much thickened. The case seems to be one of abscess of the liver ot long standing, which was probably caused by tubal trouble, following confinement, eighteen years ago.

CASE 7. Cælo-hysterectomy, for multinodular fibroid tumor. Mrs. B., age 43; very stout, weight 225 pounds; no children, no miscarriages. Has been suffering over twenty years with abdominal and pelvic pain; painful and profuse menstruation, lasting six weeks; in fact, at times, continuous hemorrhage. First noticed she was getting large fifteen years ago; has been gradually increasing in size since marriage, twelve years ago. Has been a widow four years. Has no pain now except at monthly periods, which are ragular, lasting two weeks; between the periods she suffers very much from a dragging weight. She has had all kinds of treatment, electricity, dilatation, ergot, etc. First examined her on the 14th of September, 1892. Could not reach or see the cervix, which seems to be continuous with the vagina, being able to insert my finger into the canal, which is surrounded with small fibroid growths. Can pass a sound six inches into what I suppose to be the uterus. The tumor seems to be fixed to the uterus, or rather, the tumor and uterus seem to be a mass of multinodular fibroids, and I fear if I operate I will be compelled to remove the uterus, which fact I have written to my friend, Dr. Halbert, of Waco. She is very anxious to have the operation performed, and I am as anxious to have her change her mind, on account of the thick abdominal walls and difficulty in removing the tumor and uterus, together with the fact that she has always been advised against an operation.

The operation was performad September 29th. There was an immense amount of fat, the abdominal wall being five inches thick. There were extensive adhesions to the omentum and intestines, which were torn away, and bleeding points ligated. I drew the tumor through the incision (which extended from three inches above the navel as low down as possible), and brought to view the hardest looking object I ever saw, consisting of a large fibroid tumor, attached to what I supposed to be the fundus of the uterus, which proved in turn a mass of multinodular growths. I threw a rubber tube around the mass, as low down as possible, and with scissors and fingers enucleated the uterus, making a pedicle of the peritoneal investment, having ligated the ovarian and uterine arteries, to prevent hemorrhage. I transfixed the pedicle with four large, steel knitting needles, just above the rubber tube, and closed the abdominal incision. It was impossible to stitch the peritoneal surfaces together, around the pedicle. I packed pads of borated cotton around the pedicle, and dusted the parts freely with boric acid. In order to make tension on the pedicle, and keep the parts freely together, I tied the ends of the constricting rubber tube around a large glass tube passing across the abdomen just below the pedicle, at lower angle of the abdominal incision. No drainage was used. She made an uncomplicated recovery; highest temperature 100½° F. for one day. Specimen No. 7 shows fibroids with uterus, the whole appearing as a multinodular mass.

CASE 8. Mr. H., age 38, very healthy. On the 24th of October, while hunting, he attempted to draw a Winchester rifle from the carriage, catching it by the wrong end. It accidentally discharged, the ball entering over the region of the liver, passed through his body, and came out about three inches to the right of the spine. I saw him a few hours after the accident; he was very weak from shock and loss of blood. I explored the wound as well as I could with my finger, removing some small pieces of bone, also a piece of brass. I passed a small rubber drainage tube through, and irrigated the wound thoroughly with sterilized water, covered the wounds with boric acid and borated cotton, and applied a bandage. He complained of severe pain, also pain in his right shoulder. I had to carry him six miles in an ambulance, and fifteen on the cars, which did not seem to give him much trouble. On the 27th, the pain in his side and shoulder was very severe, with symptoms of peritonitis. I opened the abdominal cavity, commencing my incision at wound of entrance, just below the eighth rib, extending the incision six inches, following the ninth rib, which was fractured in several places, with sharp points pressing against the liver. I resected a portion of the rib, when the surface of the liver was freely exposed. The ball had passed between the liver and abdominal wall, grooving the surface of the liver for about four inches. In passing my finger in the groove, I felt a hard substance, which I extracted from the liver with a pair of forceps, and proved to be a brass buckle of his suspender, carried in by the bullet. I cleansed the wound and abdominal cavity as far at I could reach, with sterilized water, removiug a large amount of clotted blood, inserted a rubber drainage tube, and closed the incision, covering all with boric acid, borated cotton, then a large pad of recently baked cotton, holding all in place with a flannel bandage. He made a good recovery, without any complications. I believe if I had not opened his abdominal cavity, he would have died.

CASE 9. Mrs. M., age 34, four children, several miscarriages. I was called to see this lady July 10th. Found her with an anxious expression, pulse 160, temperature 105° F., hurried respirations, abdomen very much distended, constantly vomiting a greenish-black fluid which her physician could not check. She had a miscarriage three weeks before, from which time her sickness dated. The physician in attendance considered the case hopeless, which, to a ce;tain extent, was correct, and advised her family to consult me. Upon examination, I believed she was suffering from acute septic peritonitis, and advised an operation to wash out and explore the abdominal cavity, which I did on the following day. As soon as the cavity was opened, there was a profuse discharge of thin, flakey pus. An abscess was found in each ovary, ruptured, the contents of which had escaped into the abdominal cavity. I removed both ovaries and tubes, washed out the cavity carefully, with sterilized water, and inserted a glass drainage tube. The temperature, in the evening, fell to 102° F., pulse 120, and there was no abdominal distension. I could not check the vomiting. To give relief, I inserted a stomach tube, removing large quantities of black fluid. She died on the 12th. This is one of those unfortunate cases where delay in making a diagnosis and resorting to proper means for relief, led to serious results; no doubt put on expectant treatment, with decomposing matters retained in the uterus. I do not think any woman is safe, after a miscarriage, until the contents of the uterus have been removed, and the organ carefully cleansed. I always wash the uterus thoroughly, after a miscarriage, with sterilized water, then peroxide of hydrogen, fifteen vol. solution, one part to three of water, using an instrument made for me by Gemrig, of Philadelphia, after a design I furnished him, having an opening in the end, and long openings just back of the end, throwing the water so as to irrigate the whole cavity. I did not expect to save this unfortunate woman's life, and the case should not be reported with my other cases of cœliotomy, thereby spoiling an otherwise good record. Still, it is of interest, and shows what may be expected after a miscarriage, under the expectant plan of treatment.

Specimen No. 7 contains the ovaries, almost completely destroyed by abscess, also enlarged tubes.

## Figures and Tables

**S. 2. f1:**
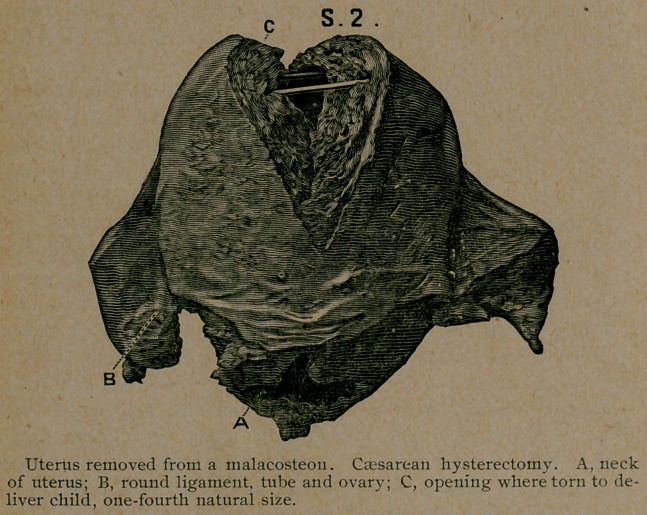


**S. 3. f2:**
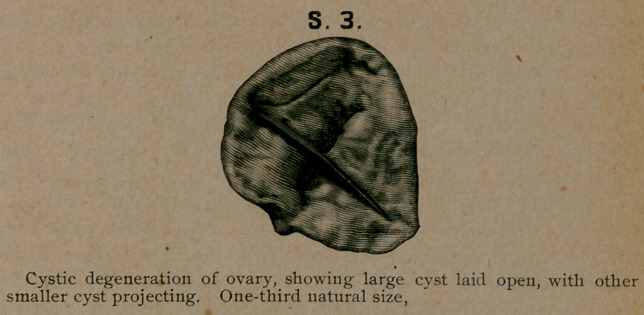


**S 6 f3:**
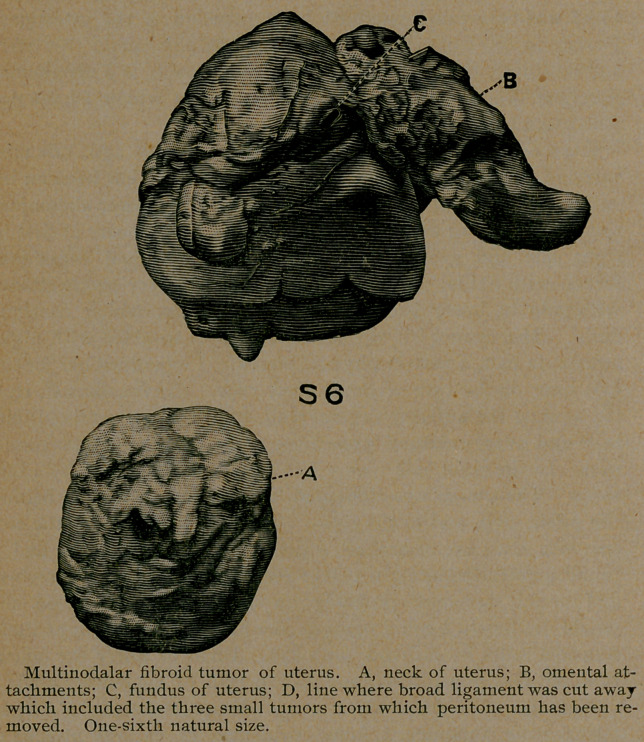


**S. 7. f4:**